# Pyramiding stacking of multigenes (PSM): a simple, flexible and efficient multigene stacking system based on Gibson assembly and gateway cloning

**DOI:** 10.3389/fbioe.2023.1263715

**Published:** 2023-11-10

**Authors:** Dongdong Zeng, Cuiyuan Jing, Lin Tang, Peng He, Jie Zhang

**Affiliations:** ^1^ The Affiliated Hospital of Southwest Medical University, Luzhou, Sichuan, China; ^2^ Department of Clinical Medicine, Southwest Medical University, Luzhou, Sichuan, China

**Keywords:** multigene stacking, Gibson assembly, gateway cloning, pyramiding stacking of multigenes, genetic engineering, synthetic biology

## Abstract

Genetic engineering of complex metabolic pathways and multiple traits often requires the introduction of multiple genes. The construction of plasmids carrying multiple DNA fragments plays a vital role in these processes. In this study, the Gibson assembly and Gateway cloning combined Pyramiding Stacking of Multigenes (PSM) system was developed to assemble multiple transgenes into a single T-DNA. Combining the advantages of Gibson assembly and Gateway cloning, the PSM system uses an inverted pyramid stacking route and allows fast, flexible and efficient stacking of multiple genes into a binary vector. The PSM system contains two modular designed entry vectors (each containing two different attL sites and two selectable markers) and one Gateway-compatible destination vector (containing four attR sites and two negative selection markers). The target genes are primarily assembled into the entry vectors via two parallel rounds of Gibson assembly reactions. Then, the cargos in the entry constructs are integrated into the destination vector via a single tube Gateway LR reaction. To demonstrate PSM’s capabilities, four and nine gene expression cassettes were respectively assembled into the destination vector to generate two binary expression vectors. The transgenic analysis of these constructs in Arabidopsis demonstrated the reliability of the constructs generated by PSM. Due to its flexibility, simplicity and versatility, PSM has great potential for genetic engineering, synthetic biology and the improvement of multiple traits.

## 1 Introduction

Recent advances in functional genomics, synthetic biology, metabolic engineering and systems biology bring powerful tools to reconstruct complex metabolic pathways and simultaneously improve multiple agronomic traits. Compared with conventional breeding, these biotechnology tools are more effective and accurate (Endy, 2005; Gibson et al., 2009; Zhu et al., 2020). Genetically modifying multiple agronomic traits and complex metabolic pathways often requires stacking two or more foreign genes in the same plant. Various strategies have been utilized for stacking multiple genes, such as iterative, co-transformation, polycistronic transgenes, plastid transformation, and multigene vector transformation (Chen et al., 2006; Dafny-Yelin et al., 2007; Bock, 2013; Zhu et al., 2020). Among the above strategies, delivering multiple genes by a multigene vector has significant advantages. By a single transformation, the multi-transgenes can be simultaneously integrated into a single chromosomal site in the host plant genome and inherited together (Chen et al., 2006; Zhu et al., 2020).

To stack multiple gene cassettes into a single T-DNA region of the binary vector, restriction enzyme-based strategies, such as Golden Gate cloning, rare cutting homing endonucleases/zinc-finger nucleases and COLORFUL-Circuit cloning, were designed and developed. Golden Gate cloning is based on type IIS restriction endonucleases and is commonly used for assembling multiple short DNA fragments (Engler et al., 2008; Goosens et al., 2021; Mukherjee et al., 2021). However, due to the high occurrence of restriction enzyme recognition sites in plant genome sequences, the capacity of Golden Gate cloning was limited for stacking DNA fragments containing multiple restriction sites (Ghareeb et al., 2016; Zhao et al., 2022). The same is true for *Sfi*I-based COLORFUL-Circuit cloning. Although rare-cutting restriction enzymes, zinc-finger nucleases and homing endonucleases-based systems have produced transformation vectors with multiple genes (Zeevi et al., 2012; Goderis et al., 2022), their applicability is limited due to the low specificity, low cutting efficiency and comparatively expensive price of the enzymes (Ghareeb et al., 2016; Zhu et al., 2020).

Exonuclease-based (or sequence overlapping-based) sequence and ligation independent cloning (SLIC) (Li and Elledge, 2007), In-Fusion (Benoit et al., 2006; Zhu et al., 2007), Gibson assembly (Gibson et al., 2009) and Stellar ExoNuclease Assembly miX (SENAX) (Dao et al., 2022) were also adopted to stack multiple DNA fragments into a binary vector. In these systems, exonucleases are utilized to produce 5′ or 3’ single-stranded overhangs. Then, the DNA fragments with homologous overhangs are joined via recombination or DNA polymerase/DNA ligase-mediated ligation. However, the application of these platforms is limited when the homologous ends have repeated sequences or stable single-stranded DNA (such as a hairpin or a stem loop). In addition, the efficiency and accuracy rate decrease when the number of DNA fragments assembled in one reaction increases (Zhu et al., 2020).

Alternatively, site-specific recombination-based Gateway cloning, homologous recombination in yeast, Cre/loxP recombination and gene assembly in *Agrobacterium* by nucleic acid Transfer using recombinase technology (GA*A*NTRY) have assembled multigene constructs. MultiSite Gateway and MultiRound Gateway are two strategies based on the commercial Gateway cloning system. The MultiSite Gateway system utilizes BP and/or LR reaction to recombine multiple fragments into a single construct in one step (Sasaki et al., 2004; Magnani et al., 2006; Wakasa et al., 2006; Kwan et al., 2007). However, the limited att sites restrict the number of genes stacked by this strategy. The MultiRound Gateway uses different entry vectors to sequentially assemble multigenes into one vector by multiple rounds of recombination reactions (Chen et al., 2006; Buntru et al., 2013). Similar to MultiRound Gateway, the GA*A*NTRY system uses A118/TP901-1 recombinase to stack multiple genes through multi-round recognition of specific attB and attP sites in *Agrobacterium tumefaciens* (Collier et al., 2018). The Cre recombinase and *loxP* site-based TransGene Stacking II (TGSII) system enables the assembly of multiple genes by Cre recombinase and irreversible *loxP* site-mediated multi-round gene stacking cycles (Lin et al., 2003; Zhu et al., 2017; Zhu et al., 2018). Large and complex multigene constructs have been achieved by MultiRound Gateway, GA*A*NTRY and Cre/*loxP* recombination. However, tedious steps are needed to construct the intermediate plasmids and to delete the bacterial resistance marker and/or donor backbone sequence after each round of recombination. The homologous recombination in yeast can assemble multiple genes in a single step, but the size of assembled molecules cannot exceed 20 kb (Shih et al., 2016). The pros and cons of the aforementioned stacking methods are listed in Supplementary Table S1. Although these methods have facilitated the development of multigene cloning techniques, more efficient and accessible DNA assembly approaches are still needed.

Combining two or more multigene stacking systems provides a new strategy for assembling multiple DNA fragments. The joint systems combine the advantages of multiple stacking systems. The recently developed TGSII-UNiE system (combined nickase-based cloning and Cre/*loxP* recombination) and GNS system (combined Golden Gate cloning and Gateway cloning) demonstrated higher stacking efficiency and flexibility than a single stacking system (Qin et al., 2022; Zhao et al., 2022). In this study, we developed a multigene vector construction system named Pyramiding Stacking of Multigenes (PSM) with the combination of Gibson assembly and Gateway cloning. The PSM system, consisting of two modular-designed entry vectors and one Gateway-compatible destination vector, can fast, flexibly and efficiently stack multiple target genes through two parallel rounds of Gibson assembly and a single-tube Gateway LR reaction. Using this system, we achieved two binary vectors stacked with four and nine gene expression cassettes, respectively. The transformation of these binary vectors demonstrated that all the transgenes existed in the leaves of transgenic *Arabidopsis thaliana*. The PSM system has great potential for genetic engineering, synthetic biology and the improvement of multiple agronomic traits.

## 2 Materials and methods

### 2.1 Microbial strains, plant materials and reagents

*Escherichia coli* strains DH5α (Transgen Biotech, Beijing, China) and DB3.1 (Tsingke Biotech, Beijing, China) were used for cloning. *Agrobacterium tumefaciens* strain EHA105 was used for Arabidopsis transformation. The vectors pCAMBIA1300 (Cambia, Canberra, Australia), pCAMBIA1301 (Cambia, Canberra, Australia), pDEST15 (Invitrogen, Carlsbad, CA, United States), pENTR-gus (Invitrogen, Carlsbad, CA, United States), pBI121-eGFP and pGEM-T easy (Promega, Madison, United States) were stored in our lab. The plasmid of pCasPA (Chen et al., 2018) was purchased from Miaoling (Wuhan, China). To clone the anthocyanin biosynthesis genes, the seed of a local black-grain rice variety, Ziyeheimi (ZYHM) was purchased from Taobao (shop id: 603507389426). The ginseng plants were purchased from Taobao (shop id: 581916862627) for the amplification of ginsenoside biosynthesis genes. The Gibson assembly kit (ClonExpress Ultra One Step Cloning Kit) was purchased from Vazyme (Nanjing, China).

### 2.2 Construction of the PSM vectors

Two entry vectors, pL1-CmRccdB-LacZ-L2 and pL3-CmRccdB-LacZ-L4, were constructed. To prepare the pL1-CmRccdB-LacZ-L2 vector, the kanamycin resistance gene of pENTR-Gus was first replaced by the ampicillin resistance gene from pGEM-T easy vector by using the ClonExpress Ultra One Step Cloning Kit, resulting in a vector named pENTR-Gus-Amp. The CmRccdB and *Lac*Z sequences were respectively isolated from pDEST15 and pGEM-T easy vectors. Then, a cloning segment (MluI-SphI-SacI-CmRccdB-NdeI-HindIII-MluI-SfiI-LacZ-SfiI-MluI) containing the fragments of CmRccdB, *LacZ* and the restriction recognition sites was synthesized by overlapping PCR. The synthesized cloning segment and pENTR-Gus-Amp backbone (including the attL1 site, attL2 site and the ampicillin resistance gene) were then amplified with 20 bp homologous ends by chimeric primers. Finally, the two fragments were ligated to generate the entry vector pL1-CmRccdB-LacZ-L2 by using the ClonExpress Ultra One Step Cloning Kit. The reaction mix was then transformed into the *E. coli* strain DB3.1. The colonies with blue color were selected on LB medium supplemented with 50 mg/L ampicillin, 50 mg/L chloramphenicol, 24 mg/L IPTG and 20 mg/L X-gal.

To construct the pL3-CmRccdB-LacZ-L4 vector, multisite-directed mutagenesis strategy (Liu and Naismith, 2008) was used to mutant attL1 site to attL3 site and attL2 site to attL4 site. The mutation of AmpR-attL1 fragment to AmpR-attL3 fragment and the CmRccdB-LacZ-attL2 fragment to CmRccdB-LacZ-attL4 fragment were introduced by two-pair synthetic primers containing the desired mutations. The AmpR-attL3 and CmRccdB-LacZ-attL4 segments were then amplified with 20 bp overlapping ends by two-pair chimeric primers. Finally, the two segments were ligated to a circular plasmid by using the ClonExpress Ultra One Step Cloning Kit, generating the pL3-CmRccdB-LacZ-L4 vector. The reaction mix was then transformed into the *E. coli* strain DB3.1. The colonies with blue color were selected on LB medium supplemented with 50 mg/L ampicillin, 50 mg/L chloramphenicol, 24 mg/L IPTG and 20 mg/L X-gal.

For construction of the destination vector pDESattR1-4, the vector backbone sequence including the hygromycin resistance gene, kanamycin resistance gene, left border T-DNA repeat, pVS1 replicon, pBR322 origin of replication and right border T-DNA was cloned from pCAMBIA1300 vector by PCR. The attR1-CmRccdB-attR2 sequence was amplified from the pDEST15 vector by PCR. The attR3 and attR4 sequences were mutant from attR1 and attR2 by PCR, respectively. *SacB* and *Lac*Z segments were respectively amplified from pCasPA and pGEM-T easy vectors. Then the attR3-SacB-LacZ-attR4 segment was assembled by overlapping PCR. Finally, the destination vector pDESattR1-4 was produced via the ligation of pCAMBIA1300 vector backbone, attR1-CmRccdB-attR2 segment and attR3-SacB-LacZ-attR4 segment by using the ClonExpress Ultra One Step Cloning Kit. The reaction mixture was then transformed into the *E. coli* strain DB3.1 and selected on LB medium supplemented with 100 mg/L kanamycin, 50 mg/L chloramphenicol, 24 mg/L IPTG and 20 mg/L X-gal. The colonies with blue color were selected. All primers used for the construction of PSM vectors are shown in Supplementary Table S2.

### 2.3 Cloning of the target gene expression cassettes

The total RNA was isolated from the leaves of ZYHM and *Panax ginseng* using Trizol reagent (Sangon, Shanghai, China) according to the manufacturer’s protocol. The cDNA was synthesized by using the PrimeScript RT reagent (Takara, Japan). The coding sequences of three anthocyanin biosynthesis genes (*OsRb*, *OsC1*, *OsDFR*) and four ginsenoside biosynthesis genes (*PgDS*, *PgPPDS*, *PgUGT74AE2*, *PgUGT94Q2*) were isolated from the cDNA of ZYHM and *P. ginseng* by PCR, respectively (Jung et al., 2014; Zheng et al., 2019). The GenBank accession numbers of these 7 genes are MK636606, MK636605, MK636607, AB265170, JN604536, JX898529 and JX898530 for *OsRb*, *OsC1*, *OsDFR*, *PgDS*, *PgPPDS*, *PgUGT74AE2* and *PgUGT94Q2*. The CaMV 35S promoters P35121 and P35cam were isolated from pBI121-eGFP vector and pCAMBIA1301 vector, respectively. The nos terminator (Tnos) and CaMV 35S ployA (T35) were amplified from pBI121-eGFP vector and pCAMBIA1301 vector, respectively. The P35121-OsRb-Tnos, P35121-OsC1-Tnos, P35cam-OsDFR-T35, P35121-PgDS-T35, P35cam-PgPPDS-Tnos, P35cam-PgUGT94Q2-T35, P35121-PgUGT74AE2-Tnos cassettes were assembled by overlapping PCR. The P35121-GFP-Tnos and P35-GUS-Tnos cassettes were amplified from the pBI121-eGFP and pCAMBIA1301 vectors, respectively. All primers used for the assembly of gene expression cassettes are shown in Supplementary Table S3.

### 2.4 Assembly of multigene constructs by PSM system

The process for assembling multiple genes by PSM system can be divided into two stages:

Stage I. Construction of the entry vectors by Gibson assembly. Apart from the different Gateway attL sites, the two entry vectors are designed with identical cloning cassettes (“SphI-SacI-CmRccdB-NdeI-HindIII” and “SfiI-LacZ-SfiI”) for accepting foreign DNA fragments. This enables the construction of the two entry vectors with same process in parallel reactions. *ccdB* and *LacZ* are used as selection markers. By two-round restriction digestions of the recognition sites flanked by *ccdB* and *LacZ*, *ccdB* and *LacZ* are removed and replaced by target expression cassettes via Gibson assembly reactions in succession. In the first round, CmRccdB segments of the entry vectors are removed by *Sph*I/*Sac*I and *Nde*I/*Hind*III digestion to linearize the entry vectors. By PCR amplification with chimeric primers, 15–20 bp homologous terminal sequences are introduced to the target gene expression cassettes. The cassettes are simultaneous inserted into the linearized vectors by Gibson assembly reactions. Then, the reaction mixtures are transformed into *E. coli* strain DH5α. Only the colonies carrying the inserted cassettes survive on the LB plates containing 50 mg/L ampicillin. The second round starts with the digestion of *Sfi*I to remove the *LacZ* segments of the intermediate plasmids acquired in the first round. By the same process as described in the first round, another one or several DNA fragments are introduced into the linearized intermediate entry vectors. The reaction mixture are transformed into *E. coli* strain DH5α and the recombinants are selected on agar plates supplemented with 50 mg/L ampicillin, 24 mg/L IPTG and 20 mg/L X-gal. The transformants carrying the desired cassettes show white color. After each round of gene assembly, *Mlu*I restriction enzyme analysis is used to identify the number and size of target genes.
Stage II. Integration the cargos into the destination vector by Gateway LR reaction. The two negative selection markers and four attR sites of pDESattR1-4 constitute two Gateway cloning cassettes (“attR1-CmRccdB-attR2” and “attR3-SacB-attR4”). By a single-tube Gateway LR reaction, the cargos from the two entry constructs are simultaneously integrated to the position of the selection markers in pDESattR1-4. Then, the reaction mixture is transformed into *E. coli* strain DH5α, and only the colonies carrying the inserted cassettes survive on the LB plates in the presence of 100 mg/L kanamycin and 8% sucrose (w/v).

### 2.5 *Escherichia coli* transformation and test for positive clone

The recombination reaction mixtures were added to *E. coli* strain DH5α/DB3.1 competent cells, and then the tubes were incubated on ice for 30 min, heated at 42°C for 90 s, again incubated on ice for 2 min. Next, 700 μL LB medium was added to the tubes and shaking at 200 rpm under 37°C for 1 h. A 100 μL aliquot of each cell suspension was spread on LB medium (agar-solidified) supplemented with specific selective markers for screening. Colony PCR was performed to test the successful assembly. Single colonies were picked with sterile pipette tips and swirled in 20 μL sterile water. Then, PCR was conducted in a 20 μL reaction volume using 0.5 μL bacteria-water suspension and PrimeSTAR Max PCR mix (Takara, Japan). The PCR program was initially by pre-denaturing at 95°C for 10 min, followed by 35 PCR cycles (10 s of denaturing, 15 s of annealing at 58°C and 15 s/kb of extension at 72°C), and a final extension step at 72°C for 5 min. The PCR products were separated by 1.5% agarose gel electrophoresis at 120 V for 20 min, and visualized using the CCD imaging system of RP2019 pgDetect (Biotech, Beijing, China). The primers used for colony PCR are provided in Supplementary Table S4. The leftover bacteria-water suspensions of positive clones were then cultured for plasmid extraction, Sanger sequencing and/or restriction enzyme digestion analysis.

### 2.6 Silver staining for verifying the insert fragments digested by *Mlu*I

Non-denaturing polyacrylamide gel electrophoresis and silver staining was conducted to detect the DNA fragments generated by *Mlu*I digestion according to Liu et al. (2017) with a few modifications. 8% non-denaturing polyacrylamide gels (mix of acrylamide and N,N′-Methylenebisacrylamide at a ratio of 29:1) were made using glass plates (10 cm × 10 cm) and a two-sided vertical electrophoresis tank (Hoefer, Halliston, United States). Then, 1.5 μL samples from each *Mlu*I digestion mixture were respectively loaded in the sample lanes. The electrophoresis was run with 0.5 × TAE buffer (20 mM Tris-HCl, 10 mM acetic acid, and 1 mM EDTA-Na_2_ at pH 8.0) at 150 V for 90 min. After electrophoresis, gels were impregnated into a AgNO_3_ solution (0.15%) for 4 min with shaking at 60 rpm, then rinsed with deionized water for 5 s and soaked in a developing solution (1% NaOH, 0.037% HCHO) until clear bands appeared. The gels were re-rinsed with deionized water for observation.

### 2.7 Arabidopsis transformation

The plasmids of pDES-4G and pDES-9G were introduced into *A. tumefaciens* strain EHA105 and subsequently transformed into wild type Arabidopsis using the floral dip method (Clough and Bent, 1998). For selection of transformants, the positive transgenic seeds were screened on 1/2 Murashige-Skoog (MS) medium with the hygromycin concentration of 30 μg/mL. The T_2_ generation transgenic plants were used for the phenotypic observation. The resistance plants were detected by PCR as described below. Genomic DNA was extracted from 100 mg fresh transgenic leaves by the CTAB method (Murray and Thompson, 1980). PCR was carried out using 50 ng genomic DNA and PrimeSTAR Max PCR mix in a 20 μL reaction. *ACT2* (*At3g18780*) was used as internal control. The PCR products were separated by electrophoresis through 2% agarose gels at 120 V for 20 min, and then visualized using the CCD imaging system of RP2019 pgDetect. The primers used for the detection of transgenes are presented in Supplementary Table S5.

### 2.8 Observation the eGFP fluorescence

The eGFP fluorescence was imaged using a Cytation 5 microscope (BioTek, Winooski, United States) with an excitation wavelength of 488 nm and emission wavelength of 525 nm. Cytation 5 software (BioTek) was used to analyze the images.

### 2.9 GUS staining assays

Histochemical GUS staining was carried out according to Jefferson (1987) with a few modifications. Briefly, the transgenic leaves were immersed in a freshly prepared reaction solution (0.2% Triton X-100, 2 mM potassium ferrocyanide, 2 mM potassium ferricyanide and 2 mM X-Gluc (5-bromo-4-chloro-3-indolyl β-d-glucuronide, sodium salt dissolved in DMSO) in 100 mM phosphate buffer, pH 7.0), vacuum infiltrated for 5 min, and incubated at 37°C for overnight. Then, the chlorophyll from the leaves was eliminated by absolute ethanol for observation.

### 2.10 Accession numbers

The sequences of the PSM vectors have been submitted to GenBank with the accession numbers OQ846952, OR217440 and OR217441 for pL1-CmRccdB-LacZ-L2, pL3-CmRccdB-LacZ-L4 and pDESattR1-4.

## 3 Results

### 3.1 The vectors of pyramiding stacking of multigenes (PSM) system

**FIGURE 1:**
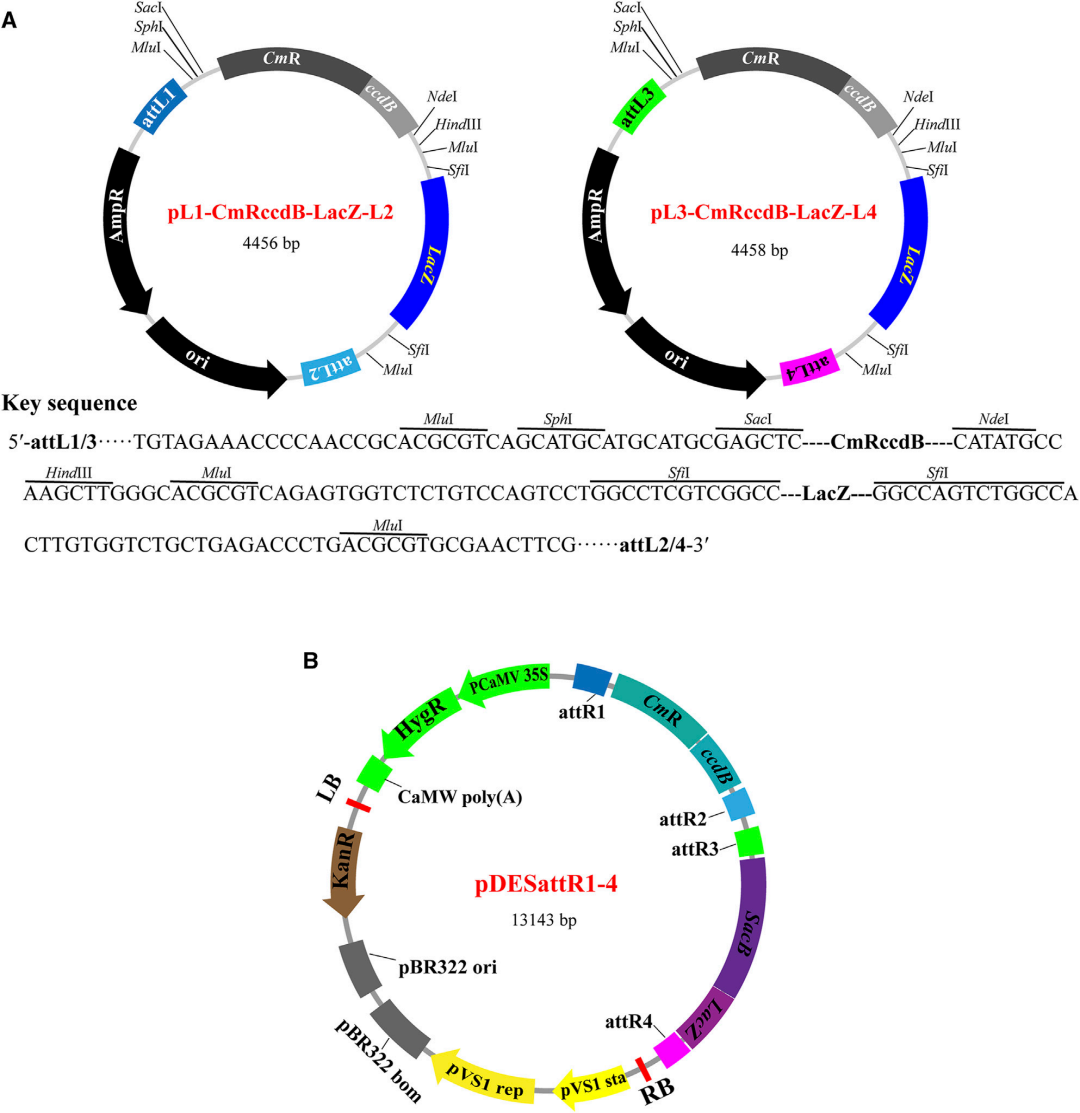
The vectors of PSM system. **(A)** Structure features of the basic entry vectors. Each of the two entry vectors has an ampicillin resistant gene, an *E. coli* replicon and a Gateway attL sited flanked segment. The attL flanked segment has two selection marker genes (*ccdB* and *LacZ*) between an attL1/attL2 pair (pL1-CmRccdB-LacZ-L2) or an attL3/attL4 pair (pL3-CmRccdB-LacZ-L4). *ccdB* is flanked by *Sph*I/*Sac*I and *Nde*I/*Hind*III recognition sites. *LacZ* is flanked by two *Sfi*I recognition sites. The product of *ccdB* is toxic to most *E. coli* strains, and only the colonies carrying target genes could survive. *LacZ* is used to screen white bacterial colonies with target genes. **(B)** Structure features of the basic binary destination vector pDESattR1-4. The pDESattR1-4 vector backbone, including the hygromycin resistance gene, kanamycin resistance gene, left border T-DNA repeat, pVS1 replicon, pBR322 origin of replication and right border T-DNA, was cloned from pCAMBIA1300 vector. *ccdB* and *SacB* (The product of *SacB* causes lethality in *E. coli* in the presence of sucrose) are used as negative screening marker genes. Two Gateway recipient regions attR1-CmRccdB-attR2 and attR3-SacB-attR4 are designed to integrate all the cargos carried by the two constructed entry vectors by a single-tube Gateway LR reaction.

The PSM system consists of two modular designed entry vectors (pL1-CmRccdB-LacZ-L2 and pL3-CmRccdB-LacZ-L4) and a binary destination vector (pDESattR1-4). The entry vectors have identical structures except for the Gateway attL sites (attL1 and attL2 in pL1-CmRccdB-LacZ-L2; attL3 and attL4 in pL4-CmRccdB-LacZ-L4) (Figure 1A). This enables cloning different genes into the two entry vectors with the same process in parallel, rather than in sequence. Each entry vector has a key component “MluI-SphI-SacI-CmRccdB-NdeI-HindIII-MluI-SfiI-LacZ-SfiI-MluI” flanked by two attL sites for accepting target DNA fragments. “SphI-SacI-CmRccdB-NdeI-HindIII” and “SfiI-LacZ-SfiI” are two cloning segments for accepting foreign gene cassettes. *ccdB* (encoding a potent poison DNA gyrase that inhibits the growth of most *E. coli* strains) and *LacZ* (for blue-white screening) are used as selectable markers to screen transformants carrying cloning products. The restriction enzyme recognition sites (*Sph*I, *Sac*I, *Nde*I, *Hind*Ⅲ and *Sfi*I) are designed for restriction digestion by corresponding restriction enzymes to linearize the vectors, thus assembling target genes by Gibson assembly reactions. After each round of gene assembly reactions, *Mlu*I restriction enzyme analysis is used to identify the number and size of the stacked genes.

The binary destination vector pDESattR1-4 is designed to accept the cargos carried by the constructed entry vectors. pDESattR1-4 has two negative selection markers flanked by four attR sites (attR1, attR2, attR3 and attR4) (Figure 1B). “attR1-CmRccdB-attR2” and “attR3-SacB-attR4” are two cloning segments to integrate the cargos carried by the entry constructs. *ccdB* and *SacB* (the product of *SacB* causes lethality in *E*. *coli* in the presence of sucrose) are used as negative selectable markers to screen target cassettes. The selectable markers carried by pDESattR1 are replaced by the cargos from the two entry constructs via Gateway LR reactions through the four attR sites in pDESattR1-4 and the four attL sites in the two entry constructs.

### 3.2 Stacking multiple gene cassettes by PSM system

**FIGURE 2:**
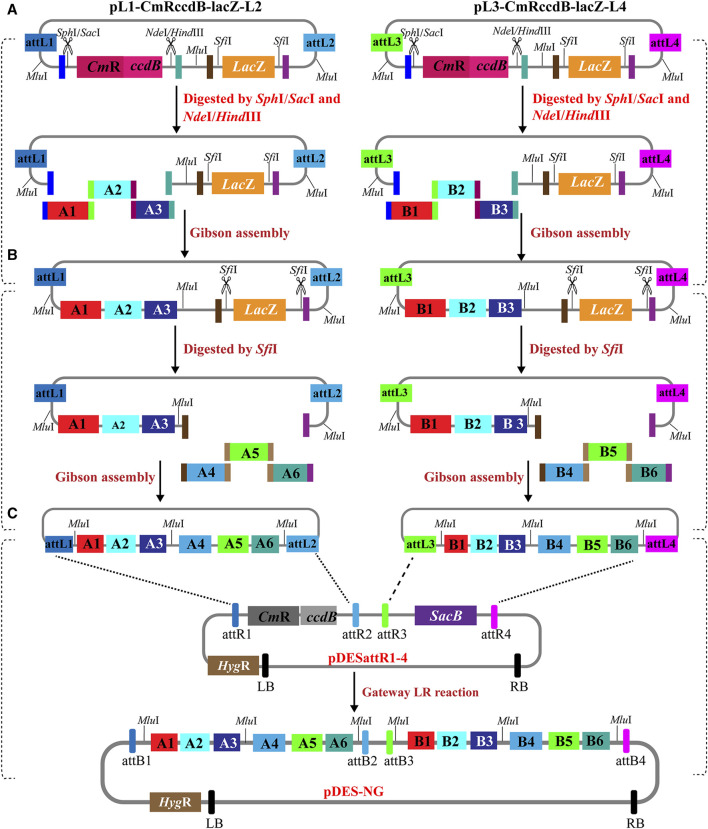
Schematic diagram of the PSM system for stacking multigenes. **(A)** The first round of Gibson assembly. The entry vectors are linearized by digestion with *Sph*I/*Sac*I and *Nde*I/*Hind*III to remove the CmRccdB cassette. One or several gene cassettes with overlapping ends are stacked into the two linearized entry vectors by Gibson assembly to get the intermediate entry vectors in parallel. **(B)** The second round of Gibson assembly. The intermediate entry vectors are linearized by digestion with *Sfi*I to remove the *LacZ* cassette. Another one or several gene cassettes with overlapping ends are stacked into the two linearized intermediate vectors by Gibson assembly in parallel. **(C)** All the target genes in the entry vectors are integrated into the destination vector by a single-step Gateway LR reaction.

The multigene stacking of PSM system is performed by two parallel rounds of Gibson assembly reactions and one-step Gateway LR reaction. The schematic representation of the stacking strategy is shown in Figures 2A–C. In the first round of Gibson assembly, the two entry vectors are linearized by restriction digestion with *Sph*I/*Sac*I and *Nde*I/*Hind*III. The gene cassettes with terminal contigs are introduced into the linearized entry vectors via parallel Gibson assembly reactions (Figure 2A). Then, the intermediate entry vectors were linearized again with the digestion of *Sfi*I. More gene expression cassettes with homologous ends are introduced into the linearized intermediate entry vectors via the second round of Gibson assembly reactions (Figure 2B). In theory, either the digestions of *Sph*I/*Sac*I and *Nde*I/*Hind*III or the digestion of *Sfi*I could be used to linearize the entry vectors in the first/second round. As *Sfi*I recognition sites occur at relatively low frequencies in the plant genomic sequences (Ghareeb et al., 2016), extra *Sph*I/*Sac*I/*Nde*I/*Hind*III recognition sites are more likely to be introduced into the intermediate entry vectors than *Sfi*I sites. If the digestions of *Sph*I/*Sac*I and *Nde*I/*Hind*III are used in the second round to linearize the intermediated vectors, these four recognition sites are more likely to conflict with the introduced ones than the sites of *Sfi*I. Therefore, the digestions of *Sph*I/*Sac*I and *Nde*I/*Hind*III are recommended to linearize the entry vectors in the first round. In the cases of the stacking cassettes carrying *Sfi*I recognition sites, these cassettes are recommended to be stacked in the second round. Finally, the gene cassettes carried by the two entry constructs are integrated into pDESattR1-4 by a one-tube Gateway LR recombination, resulting in a binary expression plasmid with multiple gene cassettes (Figure 2C). The stacking route of PSM system is like an inverted pyramid, which is more time-saving than traditional sequentially stacking strategies.

### 3.3 Generating of multigene constructs by PSM system

**FIGURE 3:**
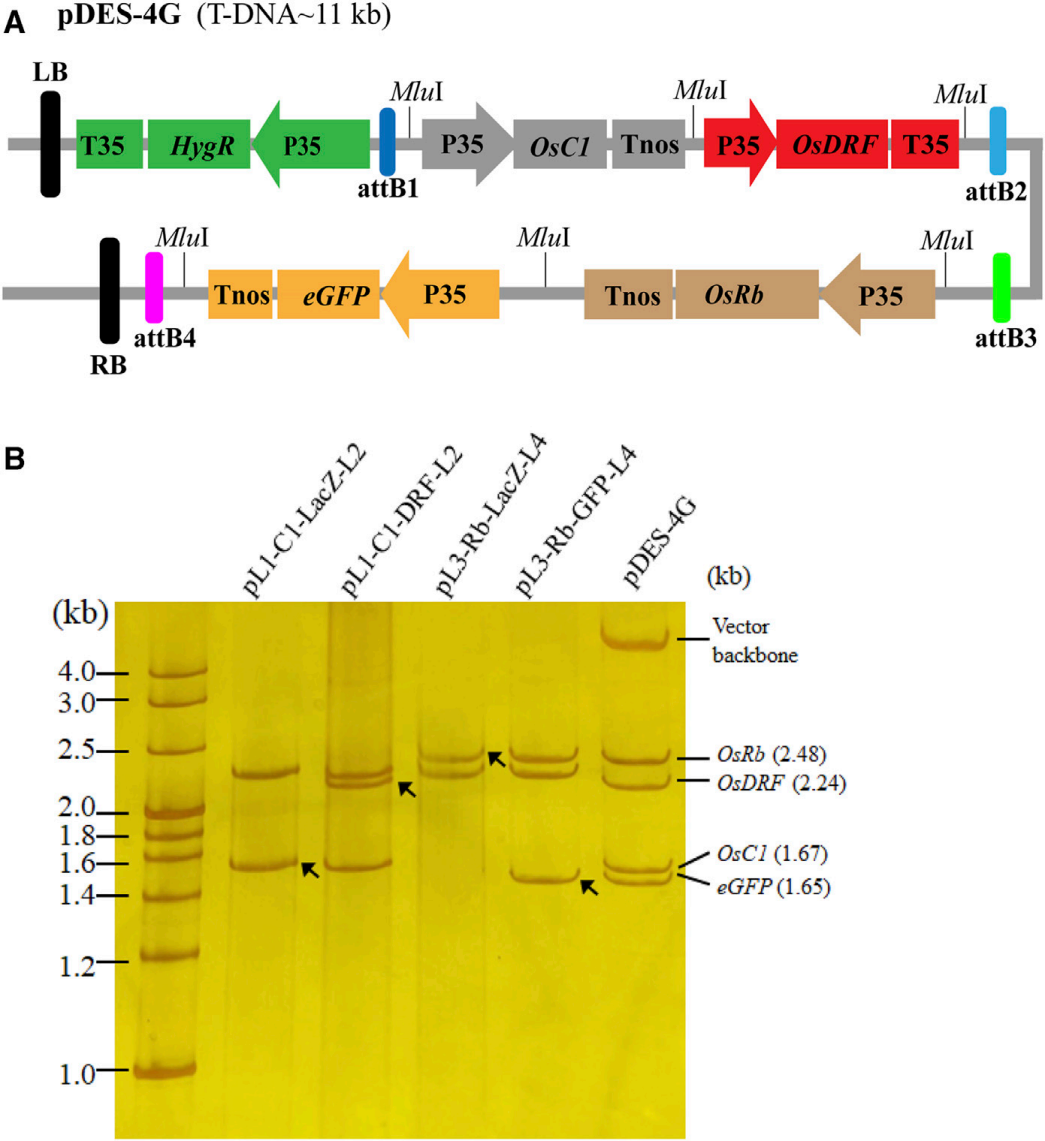
The structure and validation of the construct with four target gene cassettes. **(A)** The diagram of the four target gene-stacked T-DNA. The T-DNA contains one selectable gene and four target genes. **(B)** *Mlu*I digestion analysis of the constructs from different processed to validate the stacked genes by 8% non-denaturing polyacrylamide gel electrophoresis. The black arrows indicate the newly added gene(s) in each gene assembly round, and the red arrows refer to backbone of the entry vectors. In the lanes of pL1-C1-LacZ-L2 and pL3-Rb-LacZ-L4, there are two *LacZ* cassettes run out of the gel due to their small size (0.61 kb).

To use the PSM system, we first stacked three rice anthocyanin biosynthesis genes (*OsC1*, *OsRb* and *OsDFR*) and one reporter gene (*eGFP*) into pDESattR1-4. The schematic diagram of the vector construction process is provided in Supplementary Figure S1. By two rounds of Gibson assembly, four intermediated plasmids (pL1-OsC1-LacZ-L2, pL1-OsRb-LacZ-L2, pL1-C1-DFR-L2 and pL3-Rb-eGFP-L4) were primarily assembled. Then, the four cargos in pL1-C1-DFR-L2 and pL3-Rb-eGFP-L4 were simultaneously transferred into the binary vector pattR1-4, generating the binary multi-gene expression vector pDES-4G. The T-DNA region of pDES-4G is around 11 kb (Figure 3A). After each stacking process, 20 colonies were tested by colony PCR. The result showed that more than 19 colonies from each stacking process had the target band/bands (Supplementary Table S6). The digestion analysis of *Mlu*I confirmed the cassettes from each process of vector construction (Figure 3B).

**FIGURE 4:**
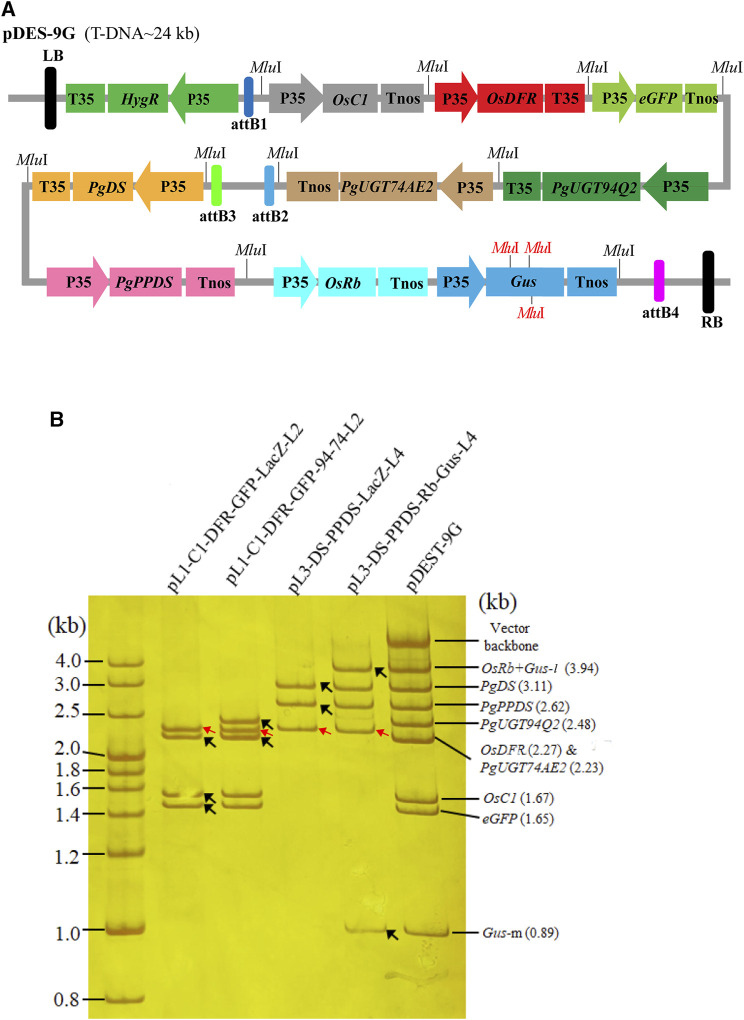
The structure and validation of the construct with nine target gene cassettes. **(A)** The diagram of the nine target gene-stacked T-DNA. The T-DNA contains one selectable gene and nine target genes. The *Mlu*I sites in red color refer to the internal *Mlu*I sites in the cassette of Gus. **(B)** *Mlu*I digestion analysis of the constructs from different rounds to validate the stacked genes by 8% non-denaturing polyacrylamide gel electrophoresis. The black arrows indicate the newly added genes in each gene assembly round, and the red arrows refer to backbone of the entry vectors. In the lanes of pL1-C1-DFR-GFP-LacZ-L2 and pL3-DS-PPDS-LacZ-L4, the LacZ cassettes run out of the gel because of their small size (0.61 kb). As the sequence of Gus cassette contains three *Mlu*I recognition sites, four fragments, namely, Gus-l (1.44 kb), Gus-m (0.89 kb), Gus-s (0.70 kb) and Gus-22 (22 bp), were produced by the digestion of *Mlu*I. Due to the small size of Gus-s and Gus-22, these two fragments run out in the gel and do not displayed in the electrophoresis image.

To demonstrate the utility of PSM system for assembling more genes, we further stacked nine gene expression cassettes into pDESattR1-4. These gene expression cassettes included four ginsenoside biosynthesis genes (*PgDS*, *PgPPDS*, *PgUGT74AE2* and *PgUGT94Q2*), three rice anthocyanin biosynthesis genes (*OsC1*, *OsRb* and *OsDFR*) and two reporter genes (*eGFP* and *Gus*). The schematic diagram outlining the steps is shown in Supplementary Figure S2. Four intermediated plasmids (pL1-C1-DFR-EGFP-LacZ-L2, pL3-DS-PPDS-LacZ-L4, pL1-C1-DFR-EGFP-94-74-L2 and pL3-DS-PPDS-Rb-Gus-L4) were primarily obtained by two rounds of Gibson assembly. Then, the nine cargos in pL1-C1-DFR-EGFP-94-74-L2 and pL3-DS-PPDS-Rb-Gus-L4 were simultaneously transferred into the binary vector pattR1-4, generating the binary multi-gene expression vector pDES-9G with a T-DNA around 24 kb (Figure 4A). The colony PCR showed that 5/20, 8/20, 6/20 and 5/20 of the colonies had all the target bands for stacking the cassettes of *OsC1*::*OsDFR*::*EGFP* (1.67 + 2.27+1.65 kb, for the construction of pL1-C1-DFR-EGFP-LacZ-L2), *PgUGT94Q2*::*PgUGT74AE2* (2.48 + 2.23 kb, for the construction of pL1-C1-DFR-EGFP-94-74-L2), *PgDS*::*PgPPDS* (3.11 + 2.62 kb, for the construction of pL3-DS-PPDS-LacZ-L4) and *OsRb*::*Gus* (2.48 + 3.02 kb, for the construction of pL3-DS-PPDS-Rb-Gus-L4), respectively (Supplementary Table S7). These results suggested that the cloning efficiency decreased when the number or the length of the cassettes assembled in one Gibson assembly reaction increased. By three replications of single-tube Gateway LR recombination with pDESattR1-4, pL1-C1-DFR-EGFP-94-74-L2 (insert size 10.48 kb) and pL3-DS-PPDS-Rb-Gus-L4 (insert size 11.47 kb), only two, three and five colonies were respectively obtained in the LB plates. The colony PCR and *Mlu*I-restriction analysis showed that all the 10 colonies had the target bands. The *Mlu*I-restriction analysis confirmed the cassettes from each process of vector construction (Figure 4B). Although one positive recombinant is enough for the experiment, the recombination efficiency for simultaneously integrating two large DNA fragments into pDESattR1-4 is needed to be improved. To increase the recombination efficiency, we took two cycles of Gateway LR reactions to separately substitute the cassettes (carried by the two entry constructs) for *ccdB* and *SacB* in pDESattR1-4. More than 100 colonies were acquired after each LR recombination cycle, and 20 colonies from each cycle were randomly selected for identification. By colony PCR and *Mlu*I digestion, 19 and 18 positive colonies were respectively identified to have all the nine gene cassettes. These results indicated that the overall stacking efficiency significantly increased by two-cycle Gateway LR recombination to separately transfer the large fragments (carried by the two entry constructs) into pDESattR1-4.

### 3.4 Plant transformation and expression analysis

**FIGURE 5:**
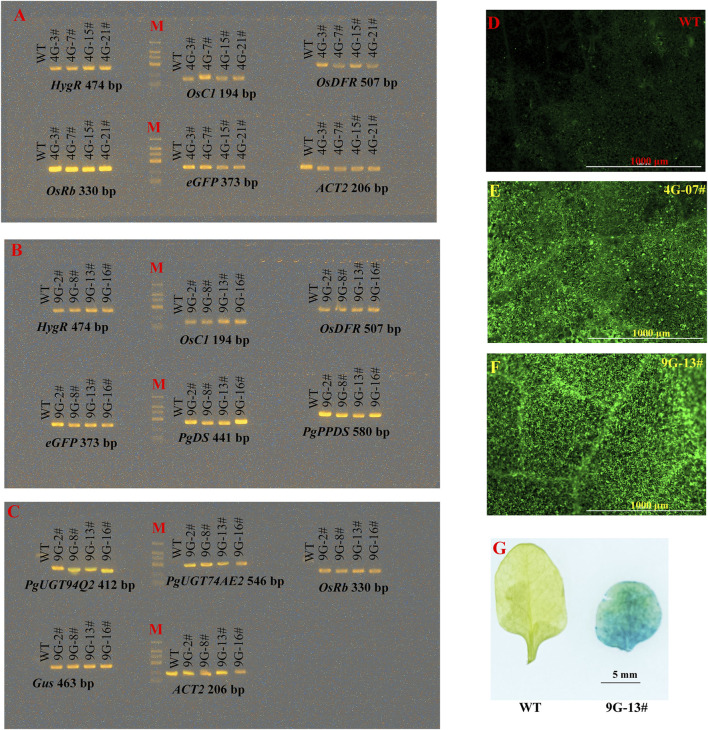
Reliability confirmation of the constructs generated by PSM. **(A)** PCR detection of the transgenes in pDES-4G-transformed Arabidopsis lines. **(B, C)** PCR detection of the transgenes in pDES-9G-transformed Arabidopsis lines. **(D–F)** The GFP signal observation of wild-type (WT), pDES-4G-transformed and pDES-9G-transformed Arabidopsis lines by fluorescent microscope, respectively. **(G)** Histochemical GUS staining of pDES-9G-transformed Arabidopsis lines.

To verify the dependability of the multigene constructs produced by PSM system, we introduced the plasmids of pDES-4G and pDES-9G into Arabidopsis via the floral dip method. By hygromycin resistance selection, 24 pDES-4G-transformed and 16 pDES-9G-transformed lines were respectively obtained. The leaves from four pDES-4G-transformed and four pDES-9G-transformed T_2_ generation lines were randomly prepared for further analysis. The PCR analysis showed that all the transgenes were positively detected (Figures 5A–C). Fluorescent microscopy examination of the GFP fluorescence in leaves from the same eight plants demonstrated the expression of *eGFP* in all the tested samples (Figures 5D–F). *In situ* GUS staining of leaves from the four pDES-9G-transformed plants revealed that *Gus* expressed in the tested samples (Figure 5G). These results verified that the multigene vectors generated by the PSM system are functional and dependable.

## 4 Discussion

### 4.1 The innovations of PSM system

In this study, we developed a new multigene stacking system named Pyramiding Stacking of Multigenes (PSM) with the combination of Gibson assembly and Gateway cloning. Using the PSM system, we assembled multigene constructs with up to ten gene cassettes (including the selection marker) by two rounds of Gibson assembly reaction and one-step Gateway LR reaction. The transgenic analysis demonstrated the dependability and reliability of the constructs generated by PSM. Combining the advantages of Gibson assembly and Gateway cloning, PSM has several innovations as described below.

First, PSM provides a simple, efficient, stable and user-friendly platform for stacking multiple genes. PSM is based on the widely used Gibson assembly and Gateway cloning. Gibson assembly and Gateway cloning have been widely used for their time and effort-saving, high cloning efficiency, and easy-to-use advantages over conventional restriction enzyme-based cloning. Although no novel cloning technology is introduced in PSM system, the easy-to-purchased commercial Gibson assembly and Gateway cloning kits are user-friendly and reliable to the researchers.

Second, PSM system has large capacity for stacking multiple DNA fragments. PSM consists of two modular-designed entry vectors and one Gateway-compatible destination vector. Since binary expression vectors usually have large size, the manipulation of these binary expression vectors is relatively less efficient. The small size of entry vectors (the backbone is only 2.2 kb) makes it easier and more efficient to stack multiple fragments. Each entry vector has two selectable makers (*ccdB* and *LacZ*) flanked by two attL sites. By sequentially removing *ccdB* and *LacZ* to linearize the entry vectors, four independent Gibson assembly reactions are allowed to clone multiple genes into the two entry vectors. Gibson assembly could assemble more than 6 DNA fragments to generate extremely large construct (more than 900 kb) (Gibson et al., 2009), so more than 24 DNA fragments could be theoretically inserted into the two entry vectors. The destination vector (pDESattR1-4) has two negative selection markers (*ccdB* and *SacB*) flanked by four attR sites. As Gateway LR reaction allows the cloning of large fragment more than 100 kb (Katzen, 2007), the cargos carried by the entry constructs could be transferred into pDESattR1-4 to produce a large construct via Gateway LR recombination.

Third, PSM is a time-saving and flexible system to stack different number of fragments via at least two rounds of stacking. The entry vectors in PSM system are modularly designed with identical structures (except the attL sites), which enable stacking different number of DNA fragments in the two entry vectors in a standardized manner in parallel, rather than in sequence. For less number (≤4) of fragments, one or two fragments could be respectively assembled into the two linearized entry vectors (by simultaneously removing *ccdB* and *LacZ* with the digestion of *Sph*I/*Sac*I and *Sfi*I) via one-round Gibson assembly. For large number (≥5) of fragments, two or more fragments could be respectively assembled into the two linearized entry vectors (by sequentially removing *ccdB* and *LacZ* with the digestion of *Sph*I/*Sac*I and *Nde*I/*Hind*III) via two-round Gibson assembly. The cargos in the entry constructs can be simultaneously delivered into the Gateway-compatible destination vector by a single-tube Gateway LR reaction. The stacking route of PSM system is like an inverted pyramid (as the schematic diagram shown in Figure 2), which saves much time and effort than the established multi-round stacking methods.

### 4.2 The advantages of PSM over Gibson assembly and gateway cloning

PSM system is derived from Gibson assembly and Gateway cloning. However, PSM has great advantages over these two methods for the assembly of multiple genes. Compared with Gibson assembly, PSM has larger loading capability, wider application range and higher cloning efficiency. Gibson assembly is a powerful technique for stacking multiple overlapping DNA molecules into a vector without relying on the availability of restriction sites (Gibson et al., 2009; Gibson, 2011). However, this system is not appropriate for the assembly of DNA fragments with repetitive sequences (such as repeated terminators and promoters). Also, the efficiency and accuracy rate decrease when the number of DNA fragments to be assembled in one reaction increases (Ghareeb et al., 2016; Liu et al., 2017). PSM system enables four independent Gibson assembly reactions to stack multiple genes into the two entry vectors. This not only increases the cloning capability of PSM, but also makes it possible to stack up to four DNA fragments with identical repetitive ends. To increase the efficiency and accuracy rate for cloning multiple genes, at most three target gene cassettes are suggested to be cloned into the entry vectors by each Gibson assembly reaction. Assuming that each Gibson assembly reaction stacks one to three cassettes, it is possible to stack 4 to 12 cassettes into the entry vectors by two rounds of Gibson assembly reactions. As the exonuclease-based methods (such as SLIC, In-Fusion and Gibson assemly) use the same types of DNA starting materials and result in the same final product (Chandran and George, 2020), PSM has the same advantages over the other exonuclease-based methods.

MultiSite Gateway and MultiRould Gateway are two Gateway-based systems for stacking multiple DNA fragments. MultiSite Gateway system utilizes BP and/or LR reaction to recombine multiple fragments into a single construct in one step (Sasaki et al., 2004; Magnani et al., 2006; Wakasa et al., 2006; Kwan et al., 2007; Petersen and Stowers, 2011). However, only four fragments could be stacked by MultiSite Gateway due to the limited att sites. In addition, the commercial MultiSite cloning kit is extremely expensive. The cost of one MultiSite Gateway reaction is 15 times higher than that of Gateway LR reaction or Gibson assembly. MultiRound Gateway uses different entry vectors to sequentially assemble multigenes into one vector by multiple rounds of recombination (Chen et al., 2006; Buntru et al., 2013). For stacking nine gene cassettes, PSM only needs three rounds of cloning (two-round of Gibson assembly and one step Gateway LR recombination), while nine rounds are needed by MultiRound Gateway. Overall, PSM has larger cloning capability and lower cost than MultiSite Gateway, and is much time-saving than MultiRound Gateway.

### 4.3 The advantages of PSM over other multigene stacking systems

Based on the stacking route, the established stacking systems could be divided to multi-round stacking systems and one-step stacking systems. For the multi-round stacking systems (such as Zinc finger nuclease and homing endonuclease-mediated assembly, TGSII and GA*A*NTRY), multiple fragments are sequentially assembled by multiple rounds of restriction enzyme digestion and ligation or site-specific recombination. Zinc finger nuclease and homing endonuclease-mediated assembly, TGSII and GA*A*NTRY have produced large and complex constructs carrying nine or even more transgenes (Zeevi et al., 2012; Zhu et al., 2017; Collier et al., 2018). However, these systems are labor-intensive and time-consuming due to the need of system-compatible intermediate vectors construction, bacterial transformation and positive transformants selection in each stacking round. For TGSII and GA*A*NTRY, extra steps are needed to delete the bacterial resistance marker and/or donor backbone sequence after each round recombination. Compared with these systems, PSM is much easy-to-operate and time-saving for stacking multiple fragments due to its inverted pyramid stacking route.

By using one-step stacking systems (such as Golden Gate cloning, COLORFUL-Circuit cloning and jStack), multiple fragments could be stacked in a single step (Werner et al., 2012; Ghareeb et al., 2016; Shih et al., 2016). The one-step stacking strategy is time-saving due to that only one stacking process is performed. However, the stacking capability of these systems is limited as the assembly efficiency decreases when the number and/or the length of fragments increase. No large plasmid with both large numbers and long length of fragments has been reported only by a single of these systems. Compared with these systems, PSM confers great stacking capacity for the assembly of large construct.

### 4.4 The limitations of PSM

As discussed above, although PSM system has advantages over the other multigene stacking systems, the cost must be taken into consideration. Unfortunately, the commercial Gibson assembly and Gateway cloning kits used in PSM are relatively expensive than the restriction enzymes and T4 ligase used in traditional restriction enzyme cloning. The cost is likely to serve as a constraint in the widely adoption of PSM. Because only three stacking rounds are used in PSM, fewer reagents are needed for the construction, transformation and selection of intermediate vectors. The cost of PSM for stacking large number of fragments is relatively low. The commercial Gibson assembly typically employs a triad of enzymes, namely, T5 exonuclease, Phusion DNA polymerase, and Taq DNA ligase. However, the cloning efficiency was not decreased even if Taq DNA ligase was removed by the modified Gibson assembly systems (Fu et al., 2014; Benoit et al., 2016). The TEDA method described by Xia et al. (2019) only uses a T5 exonuclease and has higher cloning efficiency than that of the commercial Gibson assembly kit. TEDA system is a cost-saving alternative to Gibson assembly for the labs with a budget constraint.

In conclusion, combining the advantages of both Gibson assembly and Gateway cloning, PSM is a flexible, efficient and time-saving platform for the assembly of multiple DNA fragments. By making simple modifications to the vectors, this vector system can be applicable to a wide range of organisms. The system will be a powerful tool for genetic engineering, synthetic biology and the improvement of multiple agronomic traits.

## Data Availability

The original contributions presented in the study are included in the article/Supplementary Material, further inquiries can be directed to the corresponding author.
