# Supplementary material for: Pyramiding stacking of multigenes (PSM): a simple, flexible and efficient multigene stacking system based on Gibson assembly and gateway cloning
Source: Front Bioeng Biotechnol. 2023 Nov 10;11:1263715. doi: 10.3389/fbioe.2023.1263715 (PMC10668122; doi:10.3389/fbioe.2023.1263715)
Supplement: Supplementary file 1 [file DataSheet1.docx]

Pyramiding Stacking of Multigenes (PSM): A simple, flexible and efficient multigene stacking system based on Gibson assembly and Gateway cloning

Dongdong Zeng ^1, 2^*, Cuiyuan Jing ^1^, Lin Tang ^1^, Peng He ^1, 2^ and Jie Zhang ^1, 2^

^1^ Biobank, the Affiliated Hospital of Southwest Medical University, Luzhou, Sichuan, China

^2^  Department of Clinical Medicine, Southwest Medical University, Luzhou, Sichuan, China

***** Correspondence: zengdd66@swmu.edu.cn; Tel.: +86-(0830)-3165496

# Supplementary information

**Figure S1** The schematic diagram of pDES-4G vector construction.

**Figure S2** The schematic diagram outlining the steps involved in the assembly of pDES-9G.

**Table S1** Pros and cons of the established multigene stacking systems.

**Table S2** Primers used for construction of the PSM vectors.

**Table S3** Primers used for the assembly of the gene expression cassettes.

**Table S4.** Primers used for colony PCR.

**Table S5** Primers used for the detection of the transgenes.

Table S6 Detection of the new insert/inserts in each stacking round for the assembly of pDES-4G.

**Table S7** Detection of the new inserts in each stacking round for the assembly of pDES-9G.


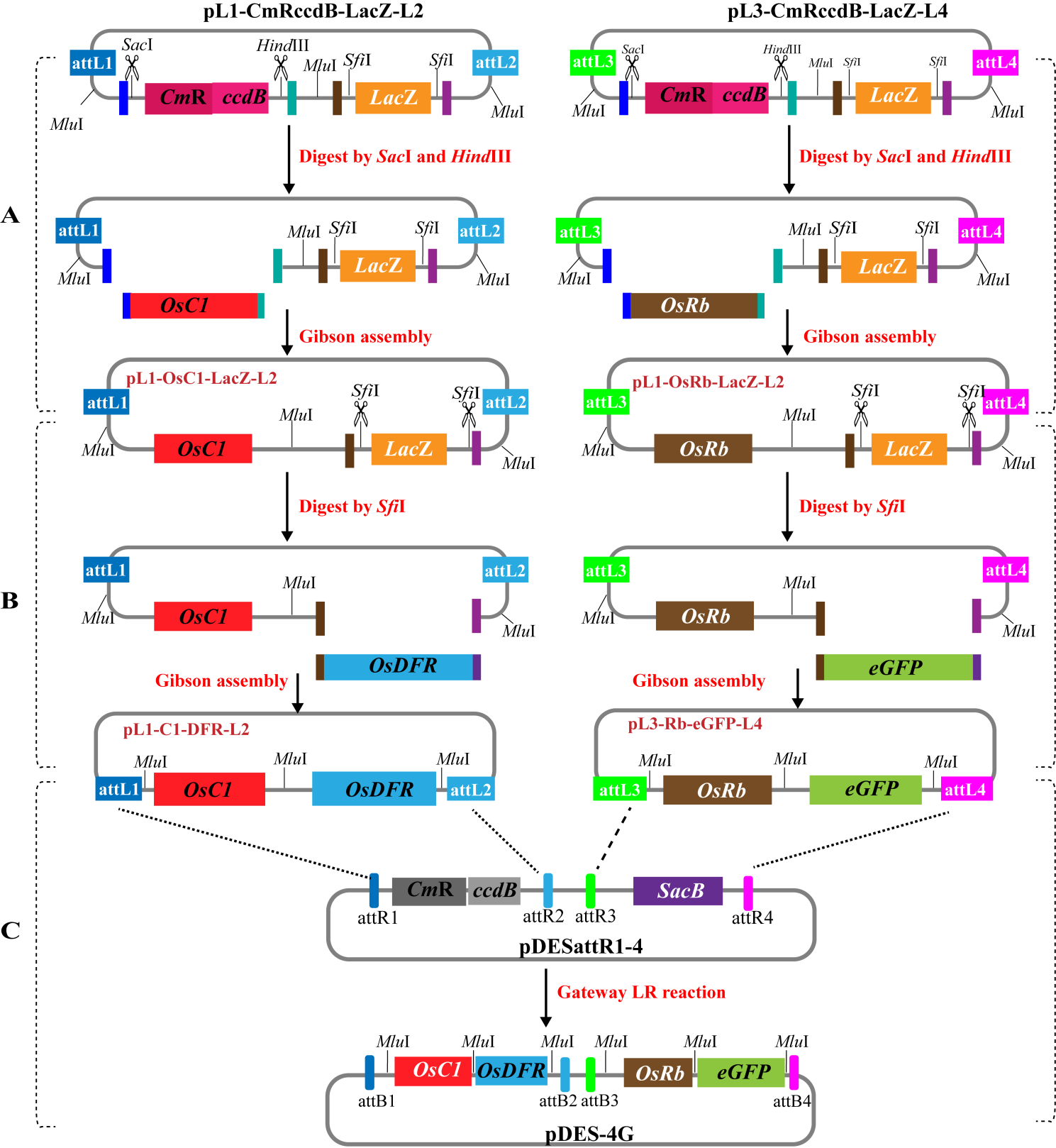


**Figure S1** The schematic diagram of pDES-4G vector construction. (A) In the first round of Gibson assembly, the CmRccdB cassettes in pL1-CmRccdB-LacZ-L2 and pL3-CmRccdB-LacZ-L4 were respectively removed by the digestion of *Sac*I and *Hind*III. The *OsC1* and *OsRb* cassettes were then cloned into the linearized vectors via Gibson assembly, yielding two intermediate entry plasmids named pL1-OsC1-LacZ-L2 and pL1-OsRb-LacZ-L2. (B) The second round of Gibson assembly began with the digestion of *Sfi*I to remove the *Lac*Z cassettes and thus linearize the intermediate plasmids. Then the Gibson assembly reactions were again applied to stack *OsDFR* and *eGFP* cassettes into the linearized pL1-C1-LacZ-L2 and pL3-OsRb-LacZ-L3, resulting in two final entry constructs named pL1-C1-DFR-L2 and pL3-Rb-eGFP-L4. (C) The cargos in the entry constructs were simultaneous transferred into pDESattR1-4 by a single tube Gateway LR reaction, generating the binary multi-gene expression vector named pDES-4G.


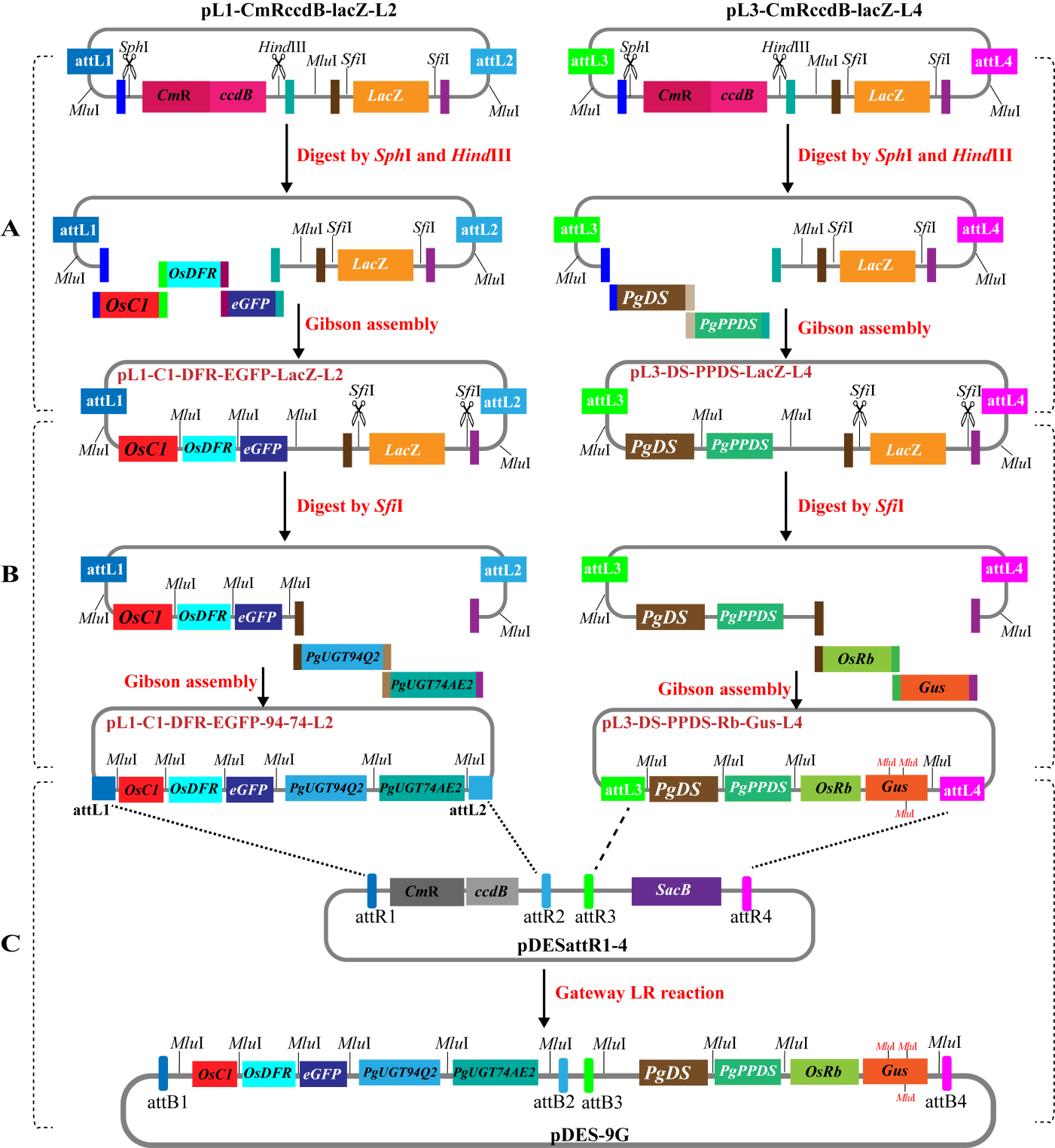
**Figure S2** The schematic diagram outlining the steps involved in the assembly of pDES-9G. (A) In the first round of Gibson assembly, the entry vectors were linearized by the digestion of *Sph*I and *Hind*III. Then, the *OsC1*, *OsDFR* and *EGFP* cassettes were introduced into the linearized pL1-CmRccdB-LacZ-L2, and *PgDS* and *PgPPDS* cassettes were cloned into the linearized pL3-CmRccdB-LacZ-L4, yielding two intermediate entry vectors named pL1-C1-DFR-EGFP-LacZ-L2 and pL3-DS-PPDS-LacZ-L4. (B) In the second round of Gibson assembly, the two intermediate plasmids were linearized by the digestion of *Sfi*I. The *PgUGT94Q2* and *PgUGT74AE2* cassettes were assembled into the linearized pL1-C1-DFR-EGFP-LacZ-L2, and the *OsRb* and *Gus* cassettes were stacked into the linearized pL3-DS-PPDS-LacZ-L4, resulting in two entry constructs named pL1-C1-DFR-EGFP-94-74-L2 and pL3-DS-PPDS-Rb-Gus-L4. (C) The cargos in the entry constructs were simultaneous transferred into pDESattR1-4 by a single tube Gateway LR reaction, generating the binary multi-gene expression vector named pDES-9G. The *Mlu*I sites in red color refer to the internal *Mlu*I sites in the cassette of Gus.

**Table S1.** Pros and cons of the established multigene stacking systems.

| **Stacking method** | **Stacking mechanism** | **Assembled size** | **Advantages** | **Disadvantages** |
| --- | --- | --- | --- | --- |
| Golden Gate cloning (Engler et al., 2009) | Digestion with type IIS restriction enzymes and ligation with T4 DNA ligase | 35 fragments/ 10-12 kb (Construct size) | i) Multi-genes are assembled in a single step; ii) Multiple fragments could be seamlessly assembled; iii) It is less expensive. | Since the restriction enzyme recognition sites occur at relatively high frequencies, extra work is needed to eliminate the internal recognition site(s). |
| Zinc-finger nucleases and homing endonucleases-Mediated Assembly  (Zeevi et al., 2012) | zinc-finger nucleases (ZFN) and homing endonucleases based digestion and ligation | 9 genes/ ~24 kb (T-DNA size) | i) It enables the modification of existing at any stage during the construction of the vector; ii) Novel ZFNs can be developed and used to stack more genes | i) The number of homing endonucleases is limited; ii) The price is relatively expensive; iii) It is time consuming to acquire ZFNS; iv) The specificity and cutting efficiency of homing endonucleases is low. |
| COLORFUL-Circuit cloning  (Ghareeb et al., 2016) | *Sfi*I digestion and the ligation of T4 DNA ligase | 4 genes/ 8774 bp (assembled genes) | i) *Sfi*I recognition sequences occur at relatively lower frequencies; ii) It is relatively inexpensive. | i) The number of assembled cassettes is limited; ii) Extra work is needed to eliminate the internal *Sfi*I recognition site(s). |
| Exonuclease-based cloning (SLIC, Gibson assembly, In-Fusion),  (Benoit et al., 2006; Li and Elledge, 2007; Gibson et al., 2009) | Exonucleases were utilized to produce single-stranded ends. Then, the DNA fragments with homologous ends are joined via recombination or ligation | 6 fragments/ 900 kb  (product size, by Gibson assembly) | i) These methods not depend on specific restriction sites; ii) The process does not introduce any undesirable scar sequences; iii) Multiple fragments can be joined in a single reaction; iv) It doesn’t require digestion and isolation DNA fragments. | i) The termini of the DNA sequence fragments to be assembled should not have stable single stranded DNA secondary structure; ii) Repeated sequences (such as the repeated terminators and promoters) are often obstacles to these methods. iii) 3. These methods are not ideal for short DNA fragments. |
| jStack  (Shih et al., 2016 ) | yeast homologous recombination *in vivo* | 4 genes/ <20 kb (T-DNA size) | Multiple genes could be assembled in a single step. | The size of assembled molecules cannot exceed 20 kb. |
| MultiSite Gateway  (Petersen and Stowers, 2011) | Gateway cloning | 4 genes/ 19.9 kb (Construct size) | The system flexibly allows the generation of constructs carrying 2 to 4 fragments. | i) The number of stacked genes is restricted by the limited att sites; ii) 3. The Gateway MultiSite kit is expensive. |
| MultiRound Gateway  (Chen et al., 2016; Buntru et al., 2013) | Gateway cloning | 8 genes/ >26kb (T-DNA size) | The assembly procedure may be repeated nearly an infinite number of times. | i) Additional *att* sites are introduced after each round of Gateway cloning; ii) Gateway cloning kit is relatively expensive. |
| TGSII (Zhu et al., 2017, 2018) | Cre/loxP recombination and  Gateway cloning | 10 genes/ ~31 kb (T-DNA size) | TGSII theoretically allows unlimited repeated assembly cycles to generate large construct. | i) The system is complicated and time-consuming; ii) Tedious steps are needed to construct the intermediate plasmids and to delete the bacterial resistance marker. |
| GA*A*NTRY  (Collier et al., 2018) | A118 and TP901-1 recombinase , ParA-MRS system | 10 genes/ 28.5-kb (T-DNA size) | GA*A*NTRY allows unlimited rounds of stacking to produce large T-DNAs and utilize native low copy and stable maintenance of the virulence plasmid within Agrobacterium. | Tedious steps are needed to construct the intermediate plasmids and to delete the bacterial resistance marker and/or donor backbone sequence after each round recombination. |

**Table S2.** Primers used for construction of the PSM vectors.

| **Primer** | **Sequence (5′-3′)** |
| --- | --- |
| **Primers for the construction of pL1-CmRccdB-LacZ-L2** | |
| L12kan-R | CTGTCAGACCAAGTTTACTCCAGAATTGGTTAATTGGTTGTAACAC |
| L12kan-R | GCGCACATTTCCCCGAAAAGTAACACCCCTTGTATTACTGTTTA |
| Amp-F | GAGTAAACTTGGTCTGACAGTTACC |
| Amp-R | CTTTTCGGGGAAATGTGCGCGGAAC |
| ccdBR-LacZF | GCGTGCCCAAGCTTGGCATATGGTGTCGACCTGCAGACTGGCTGTGTA |
| ccdbF-mlu | GCACGCGTCAGCATGCATGCATGCGAGCTCGCCAGACTACATAATACTGTAAAAC |
| LacZF-sfi | AGTGGTCTCTGTCCAGTCCTGGCCTCGTCGGCCATCTGTTACGCCGGCGGTAG |
| LacZMluHind-F | ACCATATGCCAAGCTTGGGCACGCGTCAGAGTGGTCTCTGTCCAGTCCTGG |
| LacZR-sfi | ACCGCCGGCGTAACAGATGAGTAGTCGACTAAGACGCTCACCCGC |
| LacZR2 | GGTCTCAGCAGACCACAAGTGGCCAGACTGGCCCTACCGCCGGCGTAACAGATGA |
| ccdBF-L1 | GTCCTGTAGAAACCCCAACCGCACGCGTCAGCATGCATGCATGCGA |
| LacZR3-L2 | GCGGTTTTTCACCGAAGTTCGCACGCGTCAGGGTCTCAGCAGACCACAAGTGGCCAG |
| L1gus | GGTTGGGGTTTCTACAGGAC |
| L2gus | GAACTTCGGTGAAAAACCGCAGCAGGG |
| **Primers for the construction of pL3-CmRccdB-LacZ-L4** | |
| L1F-attL3 | CATTTAACTTTAAGAAGGAGATATATACC |
| L2R-attL4 | TTATTGTTTGCCTCCCTGCTGCGGT |
| attL4F-mutation | AGCAGGGAGGCAAACAATAAACCCAACTTTTCTATACAAAGTT |
| attL3R-mutation | GGTATATATCTCCTTCTTAAAGTTAAATGGCTAACCAACTTTATTATACAAAGTTGGC |
| **Primers for the construction of pDESattR1-4** | |
| pCambiaF-cmR | GTCGAGCGCGCTTGGCTATATGGCGGGTAAACCTAAGAGAAAAGAGCG |
| pCambiaR-attR4 | TGTATGCTATACGAAGTTATGCGGTTTGCGTATTGGCTAGAGCAGCTT |
| CmRccdBF | ATAACTTCGTATAGCATACATTATACGAAGTTATTTTGGTGGTGGCGACCATCCTCC |
| CmRccdBR | GGTTATGCTAGTTATTGCTCAGCGG |
| attR3F-mutaion | GAGCAATAACTAGCATAACCTGCACGGCAACTTTGTATAATAAAGTTGAACGA |
| attR3R-mutaion | TCAGGTGCAGTAGTTCTCCTTCATAGTGACTGGATATGTTGTGTTTT |
| attR4F-mutaion | CTAGGCTGTGACTTGTGTACTAGGCTGTGACTTGTGTATTCATAGTGACTGGATATGTT |
| attR4R-mutaion | TATAGCCAAGCGCGCTCGACCAGCTAACCAACTTTGTA |
| LacZF-R3 | GGAGAACTACTGCACCTGAGAGCTCATCGGGTACCATCTGTTACGCCGGCGGTAG |
| LacZR-R4 | TTACACAAGTCACAGCCTAGGTAGTCGACTAAGACGCTCACCCGC |
| pCambiaF-SacB | TCATACTCCCGCCATTCAGAGCTTATCAGCTAAGATTACCCTGTTATCC |
| pCambiaR-SacB | AGCGGCCGCCATGGATTCTTGGCGGGTGAGCGTCTTAGTCGACTAC |
| pDNsacBF | AAGAATCCATGGCGGCCGCTGTGGATCTAGACGGCATCAGAGCAGATTGTA |
| pDNsacBR | TCTGAATGGCGGGAGTATGAAAAGTCTCGAGGCAACTTTATGCCCATGCAACA |

**Table S3.** Primers used for construction of the PSM vectors.

| **Primer** | **Sequence (5′-3′)** |
| --- | --- |
| **The cloning of *OsC1* cassette for the assembly of pDES-4G** | |
| 4G-P35-OsC1F-sac | GCGTCAGCATGCATGCATGCGACCTAACAGAACTCGCCGTAAAG |
| P35R-OsC1 | AAGGGACTGACCACCCGGGGAT |
| OsC1F-p35 | CCGGGTGGTCAGTCCCTTATGGGGAGGAGAGCTTGCTGCGCAAAGG |
| OsC1R-Tnos | CTTATCGTCGTCATCCTTGTAATCCGCACACAAGTTCCAGGCGTCGTCG |
| TnosF-OSC1 | ACAAGGATGACGACGATAAGTAAGAGCTCGAATTTCCCCGATCGTT |
| 4G-TnosR-C1-hind | TCTGACGCGTGCCCAAGCTTACGGCCAGTGAATTCCCGATCTAG |
| **The cloning of *OsDFR* cassette for the assembly of pDES-4G** | |
| 4G-P35F-sfi | AGTGGTCTCTGTCCAGTCCTGTTTGCGTATTGGCTAGAGCAGCTT |
| P35R-DFR | ATCTCATTGCCCCCCGGGATCT |
| DFR-P35 | ATCCCGGGGGGCAATGAGATATGGGCGAGGCGGTGAAGGGGCCA |
| DFR-T35 | CTTATCGTCGTCATCCTTGTAATCTTTGACCAACGCTTCTGTTTCAGC |
| T35F-DFR | ACAAGGATGACGACGATAAGTAAAGTAGATGCCGACCGGATCTGTCG |
| 4G-T35R-sfi | GGTCTCAGCAGACCACAAGTATTAACGCCGAATTAATTCGGGGGATCTGG |
| **The cloning of *OsRb* cassette for the assembly of pDES-4G** | |
| 4G-P35-RbF-sac | GCGTCAGCATGCATGCATGCGACCTAACAGAACTCGCCGTAAAG |
| P35R-Rb | AAGGGACTGACCACCCGGGGAT |
| RbF-P35 | CCGGGTGGTCAGTCCCTTATGATGGCATCTGCTCCTTCAGCT |
| RbR-Tnos | CTTATCGTCGTCATCCTTGTAATCGTAGCCTGCTACGGCTGTCCGGAG |
| TnosF-Rb | ACAAGGATGACGACGATAAGTAAGAGCTCGAATTTCCCCGATCGTT |
| 4G-TnosR-Rb-hind | TCTGACGCGTGCCCAAGCTTACGGCCAGTGAATTCCCGATCTAG |
| **The cloning of *EGFP* cassette for the assembly of pDES-4G** | |
| 4G-EGFPF-sfi | AGTGGTCTCTGTCCAGTCCTGACCTAACAGAACTCGCCGTAAAG |
| 4G-EGFPR-sfi | GGTCTCAGCAGACCACAAGTACGGCCAGTGAATTCCCGATCTAG |
| **The cloning of *OsC1* cassette for the assembly of pDES-9G** | |
| 9G-C1F-sph | AACCCCAACCGCACGCGTCAGACCTAACAGAACTCGCCGTAAAG |
| 9G-C1R-DFR | CTCTAGCCAATACGCAAACAGCAAAGACGCGTACGGCCAGTGAATTCCCGATCTAG |
| **The cloning of *OsDFR* cassette for the assembly of pDES-9G** | |
| 9G-DFRF-C1 | TGTTTGCGTATTGGCTAGAGCAGCTT |
| 9G-DFRR-GFP | TACCTATTTTCAAGTTCAGGATTAACGCCGAATTAATTCGGGGGATCTGG |
| **The cloning of *EGFP* cassette for the assembly of pDES-9G** | |
| 9G-EGFPF-DFR | CCTGAACTTGAAAATAGGTACCCACGCGTGACCTAACAGAACTCGCCGTAAAG |
| 9G-EGFPF-hind | TCTGACGCGTGCCCAAGCTTACGGCCAGTGAATTCCCGATCTAG |
| **The cloning of Os*Rb* cassette for the assembly of pDES-9G** | |
| 9G-RbF-sfi | AGTGGTCTCTGTCCAGTCCTGACCTAACAGAACTCGCCGTAAAG |
| 9G-RbR-GUS | CGCCATTCAGGCTGCGCAACACGGCCAGTGAATTCCCGATCTAG |
| **The cloning of *GUS* cassette for the assembly of pDES-9G** | |
| 9G-GUSF-Rb | GTTGCGCAGCCTGAATGGCGAATG |
| 9G-GUSR-sfi | GGTCTCAGCAGACCACAAGTCCGCCAATATATCCTGTCAAACAC |
| **The cloning of *PgUGT94Q2* cassette for the assembly of pDES-9G** | |
| 9G-P35F-94-sfi | AGTGGTCTCTGTCCAGTCCTGTTTGCGTATTGGCTAGAGCAGCTT |
| P35R-94 | ATCTCATTGCCCCCCGGGATCT |
| 94F-P35 | ATCCCGGGGGGCAATGAGATATGGATAACCAAAATGGTAGAAT |
| 94R-T35 | TCCGGTCGGCATCTACTTTATTGTTCATCTTTCTTCTTCTTACAAAT |
| T35F-94 | TAAAGTAGATGCCGACCGGATCTGTCG |
| 9G-T35R-74 | TACCTATTTTCAAGTTCAGGATTAACGCCGAATTAATTCGGGGGATCTGG |
| **The cloning of *PgUGT74AE2* cassette for the assembly of pDES-9G** | |
| 9G-P35F-94 | CCTGAACTTGAAAATAGGTACCCACGCGTGACCTAACAGAACTCGCCGTAAAG |
| P35R-74 | AAGGGACTGACCACCCGGGGAT |
| 74F-P35 | CCCCGGGTGGTCAGTCCCTTATGCTGAGCAAAACTCACATTATG |
| 74R-nos | GTTTGAACGATCGGGAGGATCTCAGGAGGACACAAGCTTTG |
| TnosF-74 | CAAAGCTTGTGTCCTCCTGAGATCCTCCCGATCGTTCAAAC |
| 9G-TnosR-74-sfi | GGTCTCAGCAGACCACAAGTGCGCGCCTTACCGGTGCTCCCGAT |
| **The cloning of *PgDS* cassette for the assembly of pDES-9G** | |
| 9G-P35-DSF-sph | AACCCCAACCGCACGCGTCAGACCTAACAGAACTCGCCGTAAAG |
| P35R-DS | AAGGGACTGACCACCCGGGGAT |
| DSF-P35 | CCCCGGGTGGTCAGTCCCTTATGTGGAAGCAGAAGGGTGCCCAAGGAAAT |
| DSR-T35 | TCCGGTCGGCATCTACTTTATTAAATTTTGAGCTGCTGGTGCTTAGG |
| T35F-DS | TAAAGTAGATGCCGACCGGATCTGT |
| 9G-T35R-PPDS | CTCTAGCCAATACGCAAACAAAGACGCGTATTAACGCCGAATTAATTCGGGGGATC |
| **The cloning of *PgPPDS* cassette for the assembly of pDES-9G** | |
| 9G-P35-DS | TGTTTGCGTATTGGCTAGAGCAGCTT |
| P35R-PPDS | ATCTCATTGCCCCCCGGGATCT |
| PPDSF-P35 | ATCCCGGGGGGCAATGAGATATGGCAGCAGCAATGGTGTTGTT |
| PPDSR-Tnos | CGGGGAAATTCGAGCTCTTAATTGTGGGGATGTAGATGAATGGGA |
| TnosF-PPDS | TAAGAGCTCGAATTTCCCCGATCGTT |
| 9G-Tnos-PDS-hind | TCTGACGCGTGCCCAAGCTTACGGCCAGTGAATTCCCGATCTAG |

The underlined sequences are the homologous ends designed for Gibson assembly reaction.

**Table S4.** **Primers used for colony PCR.**

| **Primer** | **Sequence (5′-3′)** | **Product Size (bp)** |
| --- | --- | --- |
| DSF | AGCAGAAGGGTGCCCAAGGAAAT | 1176 |
| DSR | CTCATCACCATTGGGATCTTCTGCC |  |
| PPDSF | ATGGCAGCAGCAATGGTGTTG | 1461 |
| PPDSR | TTAATTGTGGGGATGTAGATGAA |  |
| UGT94Q2F | CATGGTCACATATCTCCCTTCTTTG | 1184 |
| UGT94Q2R | AACTCCCTTGCTTTCCTCCTGATA |  |
| UGT74AE2F | ATGCTGAGCAAAACTCACAT | 1356 |
| UGT74AE2R | TCTCAGGAGGACACAAGCTTTG |  |
| EGFPF | ATGGTGAGCAAGGGCGAGGAGCTG | 717 |
| EGFPR | CTTGTACAGCTCGTCCATGCCGA |  |
| OsRbF | ATGATGGCATCTGCTCCTTCAGCT | 1609 |
| OsRbR | GTAGCCTGCTACGGCTGTCCGGAG |  |
| OsC1F | ATGGGGAGGAGAGCTTGCTGCGCAAAGG | 813 |
| OsC1R | CGCACACAAGTTCCAGGCGTCGTCG |  |
| OsDFRF | ATGGGCGAGGCGGTGAAGGGGCCA | 1116 |
| OsDFRR | TTTGACCAACGCTTCTGTTTCAGC |  |
| GusF | GGGCAGGCCAGCGTATCGTG | 463 |
| GusR | GTCCCGCTAGTGCCTTGTCCAGTT |  |

**Table S5.** **Primers used for the detection of the transgenes.**

| **Primer** | **Sequence (5′-3′)** | **Product Size (bp)** |
| --- | --- | --- |
| HygR-F | CCACGGCCTCCAGAAGAAGATGTT | 474 |
| HygR-R | TGGGGAGTTTAGCGAGAGCCTGAC |  |
| UGT94Q2-F | GGCCATAATGAAGATCCAAAAACA | 412 |
| UGT94Q2-R | CCGCCGCCAGCTTACCATTC |  |
| UGT74AE2-F | ATGCAGGCTTATATGGGAGTTGTA | 546 |
| UGT74AE2-R | CGATCCGACGTCTGCCTTGTA |  |
| Gus-F | GGGCAGGCCAGCGTATCGTG | 463 |
| Gus-R | GTCCCGCTAGTGCCTTGTCCAGTT |  |
| OsRb-F | GCTTGCTGCTGCCGTGAGGAG | 330 |
| OsRb-R | CCAGGGCGGAAGGCGTAGGTC |  |
| OsDFR-F | GCTCGTCGTCGGGCCCTTCATCA | 507 |
| OsDFR-R | GTCCCGGTCCCCCTCCCCAGTATC |  |
| GFP-F | CGTCCAGGAGCGCACCATCTTCTT | 373 |
| GFP-R | ATCGCGCTTCTCGTTGGGGTCTTT |  |
| OsC1-F | GCTGCGCAAAGGAAGGGATGAAGA | 194 |
| OsC1-R | GATGTTGCCGCGCTTGATGTTAGG |  |
| Act2-F | GCACCCTGTTCTTCTTACCG | 206 |
| Act2-R | AACCCTCGTAGATTGGCACA |  |
| DS-F | AGGTGCGTGGACTTTCTCTGAT | 441 |
| DS-R | CAAATTCCCCAAAAGCCATACCAT |  |
| PPDS-F | TCCCGAGCAGTGTTGAAAAGATG | 580 |
| PPDS-R | TGGCCGTAAGTAGCAAGTGTGAC |  |

**Table S6**. **Detection of the new insert/inserts in each stacking round for the assembly of pDES-4G.**

| **Construct** | **Insert cassette (s)** | **Test gene** | 1 | 2 | 3 | 4 | 5 | 6 | 7 | 8 | 9 | 10 | 11 | 12 | 13 | 14 | 15 | 16 | 17 | 18 | 19 | 20 |
| --- | --- | --- | --- | --- | --- | --- | --- | --- | --- | --- | --- | --- | --- | --- | --- | --- | --- | --- | --- | --- | --- | --- |
| pL1-OsC1-LacZ-L2 | OsC1 | *OsC1* | + | + | + | + | + | + | + | + | + | + | + | + | + | + | + | + | + | + | + | + |
| pL1-OsRb-LacZ-L2 | OsRb | *OsRb* | + | + | + | + | + | + | + | + | + | + | + | + | + | + | + | + | + | + | + | + |
| pL1-C1-DFR-L2 | OsDFR | *OsDFR* | + | + | + | + | + | + | + | - | + | + | + | + | + | + | + | + | + | + | + | + |
| pL3-Rb-eGFP-L4 | eGFP | *eGFP* | + | + | + | + | + | + | + | + | + | + | + | + | - | + | + | + | + | + | + | + |
| pDES-4G | OsC1+OsRb+ OsDFR+eGFP | *OsC1* | + | + | + | + | + | + | + | + | + | + | + | + | + | + | + | + | + | + | + | + |
|  |  | *OsRb* | + | + | + | + | + | + | + | + | + | + | + | + | + | + | + | + | + | + | + | + |
|  |  | *OsDFR* | + | + | + | + | + | + | + | + | + | + | + | + | + | + | + | + | + | + | + | + |
|  |  | *eGFP* | + | + | + | + | + | + | + | + | + | + | + | + | + | + | + | + | + | + | + | + |

‘+’ and ‘-’ indicate the positive and negative results for colony PCR detection of the genes, respectively.

**Table S7. Detection of the new inserts in each stacking round for the assembly of pDES-9G.**

| **Construct** | **Insert cassette (s)** | **Test gene** | 1 | 2 | 3 | 4 | 5 | 6 | 7 | 8 | 9 | 10 | 11 | 12 | 13 | 14 | 15 | 16 | 17 | 18 | 19 | 20 |
| --- | --- | --- | --- | --- | --- | --- | --- | --- | --- | --- | --- | --- | --- | --- | --- | --- | --- | --- | --- | --- | --- | --- |
| pL1-C1-DFR-EGFP-LacZ-L2 | C1::DFR::eGFP | *OsC1* | ­ + | - | + | ­­­- | - | + | - | + | - | - | + | - | + | + | - | + | + | - | + | - |
|  |  | *OsDFR* | ­- | - | + | ­­­- | - | + | - | - | - | - | - | - | + | - | - | - | + | - | + | - |
|  |  | *eGFP* | ­- | - | + | ­­­- | - | + | - | - | + | - | - | - | + | + | - | - | + | - | + | - |
| pL3-DS-PPDS-LacZ-L4 | DS::PPDS | *PgDS* | ­­­­- | - | - | + | + | - | - | - | + | - | + | - | + | + | - | + | - | - | - | - |
|  |  | *PgPPDS* | - | - | - | + | + | - | - | - | + | - | + | - | + | + | - | - | - | + | - | - |
| pL1-C1-DFR-EGFP-94-74-L2 | UGT94Q2::UGT74AE2 | *UGT94Q2* | + | - | - | + | + | - | - | - | - | + | - | - | - | + | - | + | - | + | + | - |
|  |  | *UGT74AE2* | + | - | - | + | + | - | - | - | + | + | + | - | - | + | - | + | - | + | - | - |
| pL3-DS-PPDS-Rb-Gus-L4 | Rb::Gus | *OsRb* | - | - | - | - | + | - | + | - | - | + | + | - | - | - | - | + | - | - | + | - |
|  |  | *Gus* | - | - | - | - | + | - | - | - | - | + | + | - | + | - | - | + | - | - | + | - |
| pDES-9G | C1+DFR+eGFP+DS+ PPDS+UGT94Q2+ UGT74AE2+Rb+Gus | *OsC1* | + | + | + | + | + | + | + | + | + | + | ND | ND | ND | ND | ND | ND | ND | ND | ND | ND |
|  |  | *OsDFR* | + | + | + | + | + | + | + | + | + | + | ND | ND | ND | ND | ND | ND | ND | ND | ND | ND |
|  |  | *eGFP* | + | + | + | + | + | + | + | + | + | + | ND | ND | ND | ND | ND | ND | ND | ND | ND | ND |
|  |  | *DS* | + | + | + | + | + | + | + | + | + | + | ND | ND | ND | ND | ND | ND | ND | ND | ND | ND |
|  |  | *PPDS* | + | + | + | + | + | + | + | + | + | + | ND | ND | ND | ND | ND | ND | ND | ND | ND | ND |
|  |  | *UGT94Q2* | + | + | + | + | + | + | + | + | + | + | ND | ND | ND | ND | ND | ND | ND | ND | ND | ND |
|  |  | *UGT74AE2* | + | + | + | + | + | + | + | + | + | + | ND | ND | ND | ND | ND | ND | ND | ND | ND | ND |
|  |  | *OsRb* | + | + | + | + | + | + | + | + | + | + | ND | ND | ND | ND | ND | ND | ND | ND | ND | ND |
|  |  | *Gus* | + | + | + | + | + | + | + | + | + | + | ND | ND | ND | ND | ND | ND | ND | ND | ND | ND |

‘+’ and ‘-’ indicate the positive and negative results for colony PCR detection of the genes, respectively. ‘ND’ indicates no sample detected due to that only ten colonies were obtained for the assembly of pDES-9G.
